# Working Hard and Pushing Through: A Thematic Analysis of Humanitarian Migrants’ Experiences in the Australian Workforce

**DOI:** 10.3390/ijerph182111502

**Published:** 2021-11-01

**Authors:** Patricia Cain, Alison Reid

**Affiliations:** School of Population Health, Curtin University, Kent Street, Bentley, WA 6102, Australia; p.cain@ecu.edu.au

**Keywords:** humanitarian migrant, refugee, precarious employment, integration, health, psychosocial work hazards, discrimination, bullying, resilience

## Abstract

Employment can play an important role for humanitarian migrants in their successful integration into a new country. For humanitarian migrants to Australia, there are no skill or language restrictions imposed on resettlement. Despite the benefits, humanitarian migrants often find themselves in low-status jobs and precarious working conditions. The present study examines perceptions of job quality and exposure to workforce psychosocial risk factors such as job strain, bullying, and discrimination. We conducted semi-structured in-depth interviews with 30 humanitarian migrants from South Sudan, Afghanistan, and Iraq. Thematic analysis of transcripts identified three overarching themes: Uncertainty and Insecurity, Working Hard and Pushing Through, and Positive Attitudes and Actions. Overall, our findings show that despite high levels of education and long-term residency in Australia, many of the participants struggled to find a safe and secure place in the workforce. While some spoke about their work in positive terms, their comments should not be taken as confirmation of a positive work environment. Humanitarian migrants face an uphill battle against oppressive working conditions and underemployment.

## 1. Introduction

In recent years, people migrating to Australia on humanitarian grounds have come primarily from countries in Africa and the Middle East [1] due to conflict and global displacement. Approximately 24,000 people from Sudan (later South Sudan) arrived in Australia as humanitarian migrants between the years 2000 and 2006. This group, specifically, incurred racialized negative media messaging [2] and were described as “bad refugees” because of the difficulties they faced in settling into Australia and Australian culture [3]. In addition to negative press reports, there were reports of an increased incidence in racism and discrimination directed toward people from South Sudan [4]. One explanation for the intensification of hostility has been attributed to the visibility of migrants from Middle Eastern and African backgrounds within the broader Australian community. “Visible difference” refers to the ease through which people from Africa and the Middle East are recognized within predominantly white western countries [5]. In addition, the South Sudanese came from a subsistence agricultural society which made it difficult for them to obtain skilled work in industries other than Agriculture in Australia [3]. This further restricted their integration into Australia.

A key factor in the successful integration of humanitarian migrants into their new country is employment [6]. Employment or work can be associated with a sense of belonging and self-esteem [5], as well as providing an income and the means for financial independence and well-being [7]. However, not all employment results in positive outcomes. Employment where workers are exposed to bullying and discrimination, underpayment, underemployment, or precarious work has been shown to adversely impact on psychological and physical health [8]. Under Australian immigration policy, humanitarian migrants are not resettled on the basis of skill or language ability [1], and as such, may face different or additional challenges in the labor market. Our earlier research with migrant and humanitarian migrant workers from 22 countries showed workers were exposed to a range of psychosocial risk factors, including high job strain, precarious work conditions, unfair pay, as well as bullying and discrimination [9,10,11,12]. Moreover, workers from humanitarian migrant backgrounds often find themselves in low status and low-paid jobs, despite many possessing tertiary qualifications or skills relevant to higher status jobs [13]. Yet, many humanitarian migrants show strength and resilience in facing adversities before, during, and after resettlement [7,14]. The aim of this study was to examine the perception of job quality and the workplace psychosocial stressors faced by humanitarian migrants. We were particularly interested in their interpretation and response to challenges they experience in the workplace.

## 2. Materials and Methods

Interviews were conducted with men and women from South Sudan, Afghanistan, and Iraq, aged 18 to 65, who arrived in Australian on a humanitarian visa.

Inclusion criteria: Participants had to be currently employed and able to be interviewed in English. 

Ethics approval for this study was obtained from the Human Research Ethics Committee of Curtin University.

### 2.1. Recruitment

Recruitment materials featured details on the purpose of the study, prerequisites for participation, the study procedure, ethics approval, confidentiality, and reimbursement. Material promoting the study were distributed via email and in person to a range of public and community organizations with links to migrant and humanitarian migrant populations. The study was also promoted through a dedicated Facebook page. Organizations were contacted on more than one occasion. We also employed a community leader with access to our populations of interest to approach participants on our behalf. 

### 2.2. Interviews

Participants were provided with details of the study, given the opportunity to ask questions about the project, and provided their informed consent before taking part. Semi-structured interviews ranged in length from 40 min to 1 h 20 min, were conducted by the first author, recorded, and transcribed verbatim. 

The interview topic guide was informed by survey items from the Job Quality Measure [15] previously used in three cross-sectional studies investigating psychosocial workplace stress in migrant and Australian-born workers [10]. Statements related to job security (Do you have a secure future in your job?), job complexity (Is your job complex and difficult?), job control (Do you have a lot of say about what happens?), fair pay (Do you get paid fairly for the things you do in your job?), and unfair treatment (Has there been a time in your job when you were afraid of being fired, even though you did nothing wrong?). Participants responded with a degree of agreement or disagreement. We also asked questions relating to working without pay and wage theft (Have you ever felt concerned that you have not been paid all the money you have earnt?), employment capacity (Would you prefer to work more hours if they were available?), and financial pressures, (Do you earn enough money to allow for unexpected expenses?). These questions required a yes/no response and were used to introduce the topic. Follow-up questions were based on participant response and probed frequency, impact and reaction to adverse working conditions. Demographic questions (e.g., age, occupation, educational level) were asked last. 

## 3. Approach to Analysis

The goal of the analysis was to identify themes from the conversations on workplace experience in relation to psychosocial stressors, including unfair treatment. Thematic analysis adhered to steps outlined by Braun and Clark and included “Familiarization with data, generating initial codes, searching for themes, reviewing themes, and defining and naming themes” ([16], p. 87). Recruitment and interviews occurred concurrently with the initial coding of interview transcripts. Following completion of interviews, repeated reading of transcripts and a combination of both deductive coding focused on structured question response, and inductive coding focused on follow-up conversation and examples, identified 100 codes. Codes were linked to both explicit and latent content. Codes were systematically reviewed and collapsed into three overarching themes: Uncertainty and Insecurity, Working Hard and Pushing Through, and Positive Attitudes and Actions. Within each theme, subthemes were also identified. The following analysis includes extracts from interview transcripts. To prevent potential identification of participants, extracts are labelled according to country of birth only. 

## 4. Analysis and Discussion

Thirty participants were recruited, two-thirds from South Sudan and more men (63%) than women (Table 1). The majority of participants had been living in Australia for over ten years and came from educated backgrounds. Participants worked in a range of industries in Australia, with most participants working in health care and social assistance (Table 1). 

Analysis of the interview transcripts identified the themes, insecurity and uncertainty, working hard and pushing through, and positive attributes and actions. These themes relate to the implicit ways in which participants spoke to their work experiences and represent the common threads running through responses. Within each of the themes, we explore sub themes and identify how these notions manifest through the data.

## 5. Uncertainty and Insecurity

Expressions of insecurity and uncertainly are embedded throughout participants’ comments and responses across a range of topics. Within this theme, we identify three domains where uncertainty and insecurity manifest and connect. First, language barriers. Participants spoke of insecurities around their proficiency with the English language and the relevance of the skills and abilities they gained before migrating. Second, job security. Participants reported finding the integration process frustrating, as they experienced difficulty finding secure work commensurate with their education and skills. Lastly, voicing concerns. Once in work, uncertain work arrangements and perplexing workplace relationships, perpetuated insecurity as participants struggle to negotiate their own rights and the responsibilities of others. In this theme, we also explore how experiences of insecurity and uncertainty sustain one another and function to keep workers in precarious and negative working environments.

### 5.1. Language Barriers 

Participants often spoke of the difficulties they experienced when transitioning to working in a new country and how their lack of English language skills reduced the options available to them. On arrival in Australia, humanitarian migrants are offered 510 h of English language courses [17]. However, for some, the time devoted to this practice was something they could not “afford”.

“I haven’t finished my hours yet. I did maybe 200 h and then just started looking for work because at the time, it was really challenging. I feel probably now that was the wrong choice because it could have been better if I continued … because probably now I’d be in a better job”.

(South Sudanese participant)

Participants spoke about how their lack of English skill was often misinterpreted as a lack of intelligence which compounded difficult and stressful situations.

“I don’t know sometimes. Some people they feel like the way you communicate with them, especially at the beginning, they feel like maybe you’re dumb, you don’t understand, things like that. But the fact is, you come to this a new country, it’s going to be hard, you need to learn, just put yourself in my shoes, go to another country, it’s not going to be easy”.

(South Sudanese participant)

Some participants spoke explicitly about how the language barrier limited their work opportunities and fostered insecurity when it came to pursuing educational goals.

“I got a high diploma for Accounting, but it was my English abilities, every time I found a letter pushing back. I went to sign up for the class. But if I just sit on the government money, I just run out of money, I can’t pay the rent … it was very hard, and I decided oh what for, I’d better look for another job, so I went to the factory”.

(South Sudanese participant)

“When I came, I don’t have confidence to go and continue university. Can I do it? Oh, I don’t think so—my English is not enough, this is my thought. That’s why I say to myself, just get something like aged care”.

(South Sudanese participant)

Participants considered mastery of the English language important. The combination of their insecurities over their own abilities, and other people’s uncertainty over their potential and their self-confidence. A lack of options and opportunities often led participants to pursue work beneath their capabilities or capacity, resulting in situations of over-qualification [18] and underemployment [19].

For some participants, the language of technology and the increasing reliance on computers and technology in the workplace furthered uncertainty. Not having had much experience with technology either through education or previous employment, many found themselves struggling to use the technology essential to the recruitment and training processes or for day to day tasks at work. Some felt their inexperience was putting their future at risk.

“… everything computer, so when you go to the agency yourself—he give you computer. I am 51, I don’t know much about computers. So when I go for the interview, they give you computer … but me don’t understand computer”.

(South Sudanese participant)

“We are doing our medication, everything on the computer and everything live and accidently you click that thing there—like you haven’t even given it to someone—you just click wrong thing there, you get warning … and you cannot get it back because it is live, and if you go three times [three warnings] you have to be dismissed”

(South Sudanese participant)

While some participants described situations where facilitators and co-workers offered assistance, this was limited. The more common outcome was for participants to spend additional time on tasks reliant on computer technology. As demonstrated in the extract above, this concern was particularly relevant for “older” workers. 

Language difficulties were also seen as a barrier to establishing positive relationships at work. Insecurity over language proficiency led some participants to be hesitant to communicate, often resulting in people feeling isolated and excluded. When reflecting on this, participants recognized how holding back in this way could be misconstrued as dislike for co-workers, or a deliberate attempt to segregate from others.

“The language sometimes problem, you can’t be involved with the people, sometimes I feel people avoid to speak a lot with me because of the language, yeah. Sometimes when they are using phrases and stuff for the work, it’s new to me. Even the customers sometimes, they are saying jokes sometimes, and it’s a bit hard for me to understand”.

(Iraqi participant)

“If you don’t speak English you feel intimidated, you overthink. You want to communicate with the person, but you can’t, you know, because the English is not there. And I feel some co-workers if you don’t communicate, they think maybe—this person he’s not friendly”.

(South Sudanese participant)

Having mastery of English was recognized as an advantage. Participants who reported being able to communicate clearly were able to foster secure relationships and become more involved in workplace interactions at all levels.

“At the moment, I’m happy, because the way you can reach out to people it’s easy, people will understand what you say, and you try and interact with them, everything is sweet”.

(South Sudanese participant)

Insecurity over language ability was depicted as a barrier to success in gaining and maintaining work and establishing supportive workplace relationships. The extracts presented here show how English skills enable people to be productive in the workplace and how the inability to communicate freely limits opportunities and engagement. English skills are not only important for job-specific formal communication they are also important for building workplace relationships where support and guidance may be found. Participants’ comments also raise concerns over the effectiveness of current language courses in preparing people for the workplace and the additional challenges unfamiliar technology can present. 

### 5.2. Job Security 

As participants spoke about their work experiences, they talked about the types of jobs they did and the lack of security these jobs afforded. Of the 30 participants interviewed, 15 were currently in part-time or casual employment, and four were self-employed. Participants employed casually felt particularly vulnerable.

“There is no security. I’ve been there for nearly one and a half years now, and I’m just casual, and the money’s very small and all that. How do you say that is a secure job?”.

(South Sudanese participant)

“I don’t know if maybe in the future I have a job or not—it’s not secure. I’d like a permanent job, because that’s peace of mind”.

(Afghani participant)

Due to a lack of employment opportunities and uncertainty around Australian workforce regulations, participants sometimes found themselves working in jobs where they were underpaid. Such jobs were typically acquired through unofficial channels and paid in cash.

“There was a job—people go to work in the farm. I went here and you work whole day and you come home with maybe $40, $50 and oh my lord you tired! … all the day, you leave home at five or four and you come back home six or seven and that’s all you got in your pocket. And people have to go—even when I quit—there is still a lot of people going there”.

(South Sudanese participant)

Wage theft of this nature had been found among temporary migrant workers [20]. The participants in this study reported having these experiences early on in their resettlement when they were particularly vulnerable and had limited knowledge and understanding of the workforce. In some cases, the remuneration amounts mentioned by participants represent less than half the legal casual minimum wage [21]. 

### 5.3. Voicing Concerns

As participants spoke about negative interactions and experiences at work, they expressed feelings of uncertainty and powerlessness. Participants spoke about how they felt unable to speak up when mistreated. Inaction was at times due to a lack of knowledge on how to escalate workplace concerns, and at other times, inaction was driven by financial insecurity.

“After I turned 18, I felt like I was still getting paid the rate I previously was, because once you turn 18 you get paid the adult wage, but I wasn’t really sure and I was confused, so I didn’t ask questions at the time because I wasn’t sure who to go to”.

(South Sudanese participant)

“I couldn’t raise my voice for some reason because they can email you in three days, they say look sorry we don’t have a job for you, and it’s really hard for me, because I’m in this country, I can’t get another job”.

(Afghani participant)

“I worked about 6 days and I was expecting good money, and they decided to pay $9 [per hour] instead of the $12 … wasn’t fair but at the same time you just have to keep going because it pays your bills”.

(South Sudanese participant)

Uncertainly over what to do, who to talk to, and over the consequences of one’s actions kept people in situations where they were taken advantage of. 

Responses from participants express uncertainty and insecurity across a range of domains. The comments made here suggest that participants feel unprepared to engage fully and successfully in the workforce. Language and technological abilities aside, participants were lacking in the confidence to create meaningful social connections and to challenge unfair practices. As we discuss in the next theme, migrant workers are not typically met with “open arms”, and their unfair and hostile treatment by others creates another challenge. 

## 6. Working Hard and Pushing Through

Participants often spoke of their work in terms of how hard things were for them. At the same time, they talked about how they pushed on and pushed through these hardships, sometimes with resilience and other times with resignation. In this theme, we explore the different types of hard work that participants do to support themselves and their families. The work we refer to here involves both the physical demands and the emotional labour that participants do to negotiate relationships that are sometimes discriminatory or hostile. Embedded within the discussion of challenges participants face at work were accounts of personal resolve and coping strategies, and we highlight these also. Participants expressed a strong determination to work hard and persist in the face of adversity to make better lives not only for themselves but for the family members left behind in their countries of origin.

### 6.1. Hard Work and Working Hard

Participants spoke about working in jobs that were inherently hard, and while participants made it clear that they valued hard work, and were willing to work hard, for many the jobs they were working in could be described as ‘survival jobs’ [22]. Participants persisted in hard jobs and continued working hard to provide for their families and their future.

“I work, I do anything—I do cleaning, I do kitchen hand, I do anything. I work hard in the company to survive”.

(South Sudanese participant)

“you have to work very fast, there are a lot of things to do. People can’t manage—they come work for three days, four days, and they go”.

(South Sudanese participant)

“I work three jobs, I clean at five o’clock in the morning, then seven I go to work in [] I finish three o’clock, I come home sleep two hours, then I go to [] for six hours. Why do I do that? To buy a house and put my kids in a good school, that’s why I do this”.

(South Sudanese participant)

Some pushed through taking “any” job while studying and training so they could qualify for better jobs.

“When I come here, I’m a professional cook in the hotels, so when I come to Australia, they told me you need the Australian experience—whatever you work in Europe or Africa, but we need the Australian experience. So, I say alright, my English is not good, but I will start from zero, which is kitchen hand, then I can go to my courses. So that I do for myself and I try hard to do anything to be in Australian society”.

(South Sudanese participant)

“and the labouring was a heavy job, you start six—go till six, then you run to the study and finish at nine. It was very hard”.

(South Sudanese participant)

Given the opportunity, participants would prefer to be working hard in jobs more aligned with their education, training, and skillsets. However, because of non-recognition of qualifications and a lack of opportunities, people often found themselves doing jobs beneath their education and skills, which was hard for different reasons. For some this meant the hardship of unfair pay, for others downward social mobility was hard to come to terms with. For some, this also meant having to deal with unsolicited judgement.

“It’s very hard for me, in the beginning it was very hard. I never expected to get such a job. I never expected to be a labourer, because in the past in my country I have a very good job, I was a teacher in a university, but yeah when I came here, it was very difficult for me, still I didn’t find my right position”.

(Afghani participant)

“People say—Oh you’ve got a double Masters and you’re working as a security guard—and all this, and I say—at least I’m working, and if I’m working, I work properly”.

(South Sudanese participant)

Most of our participants had been living in Australia for some time. Two-thirds had lived in Australia for 10 or more years, so many were reflecting on their early experiences in the workforce. Through their accounts of their early working experience, we got a glimpse into their integration process. For some, time had improved working conditions, although for others, despite gaining Australian experience and qualifications they still struggle to find their “right position”.

### 6.2. Working with Bullying and Discrimination 

Some participants spoke about how hurtful and unfair treatment formed part of their everyday work experience. The way participants spoke of exclusion, bullying, and discrimination reflected how hard this was to endure, and the emotional labor required to negotiate such environments. As the following extracts demonstrate, co-workers and supervisors were both identified when participants spoke about negative encounters.

“He [a co-worker] made me cry. He was saying you are from Afghanistan, yeah there are all Taliban’s—and saying bad things about my country”.

(Afghani participant)

“I work in a place where the majority of people they are from the same country, so I’m the only one there, some of them if I am rostered with them, they will alter their roster”.

(South Sudanese participant)

“My first job was the tough one, the supervisor was rude, if I look at it now, I feel like I’ve been bullied, but at the time, I was afraid to talk, afraid to do something about it. The supervisor was really bad—you know what I mean”.

(South Sudanese participant)

As participants spoke of these events, they spoke of how they responded, or in some instances did not respond to bullying and discrimination. The South Sudanese participant in the extract above confesses to “being afraid to talk” and endures a hostile working environment. Managing hostile or dismissive relationships in ways that did not jeopardize job security meant that responses needed to be well considered. In a response similar to that described in the previous theme “voicing concerns” this was often achieved through “keeping quiet” as the following extracts attest.

“It was really tough, it was really hard—like you feel sometimes before you go to work you are happy, but when you get there, you’re not happy at all. You’re doing it because you have that fear, if I lose this job, I’m not going to get another one. I just keep silent, whoever says, whatever—I just keep quiet”.

(South Sudanese participant)

“You need to just move on with it, you can’t do much about it, because if you want to try to get into a fight with everyone at the end of the day you going to be in trouble, so it’s better just suck it in and then just let it go—you know what I mean”.

(South Sudanese participant)

Participants’ abilities to sustain themselves within adverse working environments appeared driven by fear of losing ones’ job. Given that many participants had spoken about long periods of unemployment, these fears were founded in experience. The above extracts also indicate that continuing to work in environments where one was victimized was inherently difficult and the decision to “push through” came at a cost to wellbeing. One participant’s ability to persist in this situation was recognized by other workers.

“I’ve got friends, one from Africa, one from India, we started together, we did the induction together, but them when they see the treatments not right, they just left. But I just kept working—but at the end they [established employees] ask me like—how come you’re still here? I just tell them—I’m here to work, I come here and do my job and that’s it”.

(South Sudanese participant)

For some, working in environments where they did not feel welcomed or included made already difficult and demanding jobs even harder. Participants were missing out on the support and guidance typically found in co-worker relationships. As the previous extract suggests, unfair treatment does not go unnoticed by others, and comments such as these allude to the socially accepted nature of bullying and discrimination in some workplaces.

In addition to reports of unfair treatment from co-workers and supervisors, some participants reported adverse treatment from clients. Support workers in the aged care industry were particularly at risk here. As the following extracts demonstrate, this was often the experience for South Sudanese participants.

“Normally the elderly people they never come closer with the people with the darker skin like this. Once you go there to talk to her, if you go there, she will warn you from far away—don’t touch me, you so dark, you look ugly, don’t come closer”.

(South Sudanese participant)

Some of the residents I can clearly say they are racist—they don’t like the dark people around” (South Sudanese participant).

Again, participants pushed through these situations. As the following extracts demonstrate, sometimes this was with the support of co-workers. At other times participants did the hard work of attempting to understand and rationalize client behavior, often making concessions for negative treatment.

“At first, I thought it was bad, sometimes I’d go and sit and cry and cry and then, yeah you’ve got some of the colleagues, they go and talk to you nicely, and the manager will come and talk to you nicely, and give you the advice—that’s how they are—you just take it easy”.

(South Sudanese participant)

“… you get very stressed sometimes, but when you come back and you go back to that person’s condition … when they tell you their story—they never have brown skin people working with them before”.

(South Sudanese participant)

“Discrimination only because of the clients, but for me, I can see it’s their rights, whatever they want, it’s their choice”.

(South Sudanese participant)

“Sometimes, if someone tells you something—make you angry—so you try and make it like—Ok he say that, but maybe he just like, he doesn’t understand”.

(South Sudanese participant)

Humanitarian migrants are recognized as facing challenges in finding work that is commensurate with their skills. As we find here they are often segregated into low paying, low status jobs, such as aged care [13]. Participants here worked physically hard and they also worked with people who made their work places emotionally difficult. In recent years Australia has seen an increase in racial discrimination toward people from Africa [5], and this is evidenced here also. Where discrimination results in a lack of social support, participants draw upon their resilience to interpret adverse events to push through these situations.

## 7. Positive Attitudes and Actions

Participants often reflected on their personal qualities and attributes. Some qualities enabled participants to deal with the situations they faced at work, giving them the ability and the perspective to push through the uncertain and challenging times they were facing. While we discuss what may be considered positive experiences and outcomes through this theme, it is important to keep in mind that these experiences occur in relation to significant challenge and hardship. The ability of the participants to “look on the bright side” is repeated throughout this theme.

### 7.1. Strong Work Ethic

In the previous theme, we focused on the hard jobs participants found themselves in and the challenges these presented. While participants discussed how hard some of their jobs were or had been, participants also expressed a willingness to work hard. Participants often referred to their inherent strong work ethic, an attribute central to their ability to manage and succeed in the workplace.

“That is because when I take a job, I really work. I work properly. I’ve got certificates from getting the award for the worker of the month and all this type of stuff. Wherever I work, I will really work. I really put my time to work, I don’t play around—I really do it”.

(South Sudanese participant)

Participants also talked about the genuine pride they took in their work, and the inherent value found in the work they did. This was particularly the case for people working in support roles.

“If someone waiting for you—and you have to shower them—or you have to feed them—I feel like I am doing a good job. I feel I’m important and I’m proud what I’m doing”.

(South Sudanese participant)

“I feel happy supporting people with disability, and you know help their needs. I feel happy that I’m helping—not because of money—but because I have the passion to help the people”.

(South Sudanese participant)

The way participants spoke of their willingness to do hard work demonstrated an intrinsic motivation and desire to do a good job. Participants did not frame this talk in a narrative of attempting to “prove themselves worthy” rather, they spoke of these qualities as being a natural attribute and something they were proud of. For people working in social support and caring roles, jobs typically considered low status, there was no indication that participants considered these jobs to be lacking in any way. While participants sometimes referred to caring jobs as challenging due to the racist comments and actions of clients, there were similarly declarations of pride and satisfaction with this work.

### 7.2. Resilience

When discussing experiences of unfair treatment, participants sometimes followed up with explanations of how these adverse events did not negatively impact their wellbeing or reduce their self-efficacy. The tendency to “push through” in the face of hard work and insecure working conditions has already been highlighted. In this theme, we bring attention to how resilience is framed as an inherent trait. As mentioned previously, participants working in support services frequently faced overt racism from clients. However, some faced this treatment with a strong confidence in their self-worth and ability to perform the role.

“I don’t have to take their issue as a big deal for me—that’s why if the client say they don’t like maybe because I’m black or from Africa, for me it’s not a big deal. I’m African yeah—god create me like that—what you gonna do? There is others that can help you—I will help others too—it’s not a big deal for me”.

(South Sudanese participant)

“If they send you to someone—and they are like I don’t want to see you—you black Africans … they don’t like me—it’s ok—I am here to help. They don’t like my help, let him wait for someone that he like it. But I am sure if I help him, it will be better than someone else. I know myself, I can do my job 100%”.

(South Sudanese participant)

Even when unfair treatment at work was depicted as difficult at the time, some recalled negative work environments as resilience building and preparation for future challenges.

“But I came out from there—I was like ok, I like handled, I felt like once I got out from that job, and then I didn’t quit, after a while I find another job and I left, but I felt like that was the turning point for me, I felt like ok—now if I succeeded in this job—I think anywhere in Australia if I work with whoever, I can succeed, you know what I mean. I work there for three years even though it was tough and that person was really bullying—I stay there”.

(South Sudanese participant)

When contextualizing adverse work-related events, some participants reflected on difficult lives in their home countries, referring to this as a basis for why they are able to endure unfair treatment and hardships here.

“When your life has always been struggling you tend to—you know—take things easy. So even if there’s no money I don’t really get much like stress. ‘Cos where I come from, its challenge we don’t even have much … like here at least you can see there is something in the fridge, but sometime like back home I remember we can go even up to, let me say, two days no food”.

(South Sudanese participant)

“I was born in South Sudan, I grew in Congo … I’ve seen a lot. I’ve learnt a lot also”.

(South Sudanese participant)

“Before coming [to Australia] I didn’t think about a job, nothing, the bad situation was lack of security in my country—so I was thinking about my life!”.

(Afghani participant)

Interviews with humanitarian migrants from the former Yugoslavia, the Middle East and Africa, living in Australia, [23] identified a paradox where participants reported both high levels of discrimination and high levels of positive wellbeing, and our findings here concur. In this current study, participants reported on how negative events did not impact adversely on their self-esteem. Moreover, at times, they gained strength from them. In the face of oppressive working conditions, many participants expressed integrity and resilience; confident in their ability to withstand their current working conditions and to find positives where possible. Extracts demonstrate how resilience and personality factors play an important role in mitigating the negative effects of intolerance. Reporting an ability to draw strength from previous hardships similarly aligns with McIntosh’s “earned strength” [24,25]. As participants make comparisons to their previous hardships, their current hardships seem less impactful.

## 8. Conclusions

This study set out to learn more about how humanitarian migrants experience the Australian workplace. We focused on exposure to psychosocial stressors at work, including precarious work, job complexity, unfair pay, and unfair treatment, and how they meet and respond to those challenges. Qualitative analysis revealed three key themes, Uncertainty and Insecurity, Working Hard and Pushing Through, and Positive Attitudes and Actions. Findings show how despite bringing education and experience to Australia, many humanitarian migrants are working hard in low paid insecure employment, where they regularly face racism and bullying. Despite difficult working conditions, participants spoke about how they pushed through these conditions out of necessity to provide for their family here and those left in their home country. The participants expressed how they felt little recourse over unfair treatment; this was at times due to language barriers or lack of workplace knowledge. However, lack of action was mostly attributed to feeling insecure in their employment.

Our findings echo earlier Australian research on humanitarian migrants [5,13,23,26] indicating little has changed in the past decade. Unlike Colic-Peisker [13] who employed bilingual interviewers, all our participants were interviewed in English. As much as possible, this strategy was to mediate the effect lack of language skill may have on participants’ workplace experiences. Despite long term residency in Australia and high levels of education, many humanitarian migrants are still experiencing underemployment, precarious working conditions, and discrimination.

Notes

Transcription conventions are as follows: [] indicates section of transcript excluded for reasons of confidentiality; [with text] works inserted by researcher for context/clarification; … pause in talk.

## Figures and Tables

**Table 1:** Participants’ socio demographic and employment characteristics (*n* = 30).

Participant Characteristics	*n* (%)
Country of birth	
South Sudan	20 (67)
Afghanistan	8 (27)
Iraq	2 (6)
Male	19 (63)
Female	11 (37)
Age range (years)	
18–25	5 (17)
26–35	10 (33)
36–45	9 (30)
46–55	6 (20)
Duration of residence in Australia	
0–5 years	6 (20)
5–10 years	5 (17)
10+ years	19 (63)
Highest educational attainment	
High school	5 (17)
Certificate/diploma	11 (37)
Trade/apprenticeship	1 (3)
Bachelor’s degree or higher	13 (43)
Employment status	
Casual	9 (30)
Part-time	6 (20)
Full-time	11 (37)
Self Employed	4 (13)
Industry of employer	
Construction/Trade	2 (6)
Food services	9 (30)
Education and training	2 (6)
Health care and social assistance	10 (33)
Cleaning	2 (6)
Warehousing	2 (6)
Other	3 (10)

## Data Availability

All requests to A/Prof Alison Reid—alison.reid@curtin.edu.au.
